# Supplementary material for: Complexome profiling of the Chlamydomonas psb28 mutant reveals TEF5 as an early PSII assembly factor
Source: Plant Cell. 2025 Mar 17;37(6):koaf055. doi: 10.1093/plcell/koaf055 (PMC12164590; doi:10.1093/plcell/koaf055)
Supplement: koaf055_Supplementary_Data [file koaf055_supplementary_data.zip › psb28_interactive.zip!/plots/intensity/qualityPlots/peptides/psbB_Cre-1.g2717002.t1.p1.html]

### Cre-1.g2717002.t1.p1

Trivial name: psbB  
SeqCoverage [median]: 40.60  
Mass: 56.10 kDa  
  
 

| Sequence | MissedCleavage | Mass [Da] | wtIntensity | muIntensity | Score |
| --- | --- | --- | --- | --- | --- |
| ADVPFR | 0 | 703.37 | 1350730.83 | 109962.54 | 177.42 |
| ADVPFRK | 1 | 831.46 | 140125.55 | 7611.42 | 111.73 |
| AQLGEIFEFDR | 0 | 1323.65 | 347618.95 | 38208.36 | 252.68 |
| AQLGEIFEFDRSTLQSDGVFR | 1 | 2414.19 | 17164.83 | 0.00 | 105.32 |
| DQEGRELFVR | 1 | 1247.63 | 94414.88 | 8537.72 | 112.13 |
| DVFAGIDDDINDQVEFGK | 0 | 1995.91 | 395250.00 | 0.00 | 229.32 |
| DVFAGIDDDINDQVEFGKYK | 1 | 2287.06 | 12432.27 | 0.00 | 141.60 |
| ELFVRR | 1 | 818.48 | 33902.68 | 0.00 | 142.63 |
| GLPWYR | 0 | 790.41 | 117827.51 | 21996.72 | 102.29 |
| IPEKLAFYDYIGNNPAK | 1 | 1952.00 | 156861.72 | 24193.65 | 184.37 |
| KAQLGEIFEFDR | 1 | 1451.74 | 1223426.71 | 200504.51 | 210.31 |
| KAQLGEIFEFDRSTLQSDGVFR | 2 | 2542.28 | 89453.40 | 0.00 | 138.35 |
| KLGDTSSLR | 1 | 975.53 | 293952.33 | 0.00 | 218.73 |
| KLGDTSSLREAF | 2 | 1322.68 | 91033.07 | 11373.93 | 145.31 |
| LAFYDYIGNNPAK | 0 | 1484.73 | 2536206.18 | 301906.81 | 310.21 |
| LAFYDYIGNNPAKGGLFR | 1 | 2015.03 | 1545.05 | 0.00 | 82.10 |
| LGDTSSLR | 0 | 847.44 | 1813887.37 | 201260.71 | 244.80 |
| LGDTSSLREAF | 1 | 1194.59 | 698383.85 | 94818.17 | 214.71 |
| MPTFFETFPVLLLDK | 0 | 1796.94 | 37621.12 | 0.00 | 262.56 |
| MPTFFETFPVLLLDKDGIVR | 1 | 2337.24 | 204049.50 | 0.00 | 267.38 |
| QGMFVLPFMTR | 0 | 1325.66 | 1132361.04 | 47619.14 | 169.99 |
| RMPTFFETFPVLLLDK | 1 | 1953.04 | 93406.38 | 0.00 | 142.39 |
| RMPTFFETFPVLLLDKDGIVR | 2 | 2493.35 | 334625.03 | 0.00 | 163.42 |
| RVQASLAEGASLSDAWSR | 1 | 1902.95 | 36739.16 | 0.00 | 107.78 |
| STLQSDGVFR | 0 | 1108.55 | 2452742.07 | 153316.96 | 284.35 |
| TALDLPK | 0 | 756.44 | 1076001.99 | 45458.24 | 163.34 |
| TGAMNSGDGIAVGWLGHASFK | 0 | 2074.99 | 213571.62 | 7466.28 | 301.51 |
| TGAMNSGDGIAVGWLGHASFKDQEGR | 1 | 2660.24 | 197029.63 | 5397.64 | 187.72 |
| TIFRDVFAGIDDDINDQVEFGK | 1 | 2513.21 | 13482.36 | 0.00 | 127.80 |
| VHTVVINDPGR | 0 | 1205.65 | 1380259.94 | 41547.70 | 192.38 |
| VQASLAEGASLSDAWSR | 0 | 1746.85 | 284142.67 | 4917.42 | 310.57 |
| VQASLAEGASLSDAWSRIPEK | 1 | 2214.13 | 3320.06 | 0.00 | 46.83 |
| YQWDQGFFQQEIQK | 0 | 1843.85 | 357690.49 | 7430.56 | 266.93 |
| YQWDQGFFQQEIQKR | 1 | 1999.95 | 308674.62 | 49275.65 | 212.43 |
|  |  |  | 17539934.9 | 1382804.1 |  |
